# The role of tripartite motif-containing 28 in cancer progression and its therapeutic potentials

**DOI:** 10.3389/fonc.2023.1100134

**Published:** 2023-01-23

**Authors:** Yiqing Yang, Shiming Tan, Yaqian Han, Lisheng Huang, Ruiqian Yang, Zifan Hu, Yi Tao, Linda Oyang, Jinguan Lin, Qiu Peng, Xianjie Jiang, Xuemeng Xu, Longzheng Xia, Mingjing Peng, Nayiyuan Wu, Yanyan Tang, Xiaoling Li, Qianjin Liao, Yujuan Zhou

**Affiliations:** ^1^ Hunan Key Laboratory of Cancer Metabolism, Hunan Cancer Hospital and the Affiliated Cancer Hospital of Xiangya School of Medicine, Central South University, Changsha, Hunan, China; ^2^ University of South China, Hengyang, Hunan, China; ^3^ Hunan Key Laboratory of Translational Radiation Oncology, Changsha, Hunan, China

**Keywords:** TRIM28, E3 ligase, proliferation, EMT, stemness sustainability, immune regulation

## Abstract

Tripartite motif-containing 28 (TRIM28) belongs to tripartite motif (TRIM) family. TRIM28 not only binds and degrades its downstream target, but also acts as a transcription co-factor to inhibit gene expression. More and more studies have shown that TRIM28 plays a vital role in tumor genesis and progression. Here, we reviewed the role of TRIM28 in tumor proliferation, migration, invasion and cell death. Moreover, we also summarized the important role of TRIM28 in tumor stemness sustainability and immune regulation. Because of the importance of TRIM28 in tumors, TIRM28 may be a candidate target for anti-tumor therapy and play an important role in tumor diagnosis and treatment in the future.

## 1 Introduction

Tripartite motif-containing (TRIM) protein family contains more than 80 members, which are involved in a variety of cellular processes and signal pathways (1, 2). The common feature of TRIM family is that they contain an amino (N) terminus TRIM structure, also known as the RBCC domain, which contains a RING (Really Interesting New Gene) finger structure (which may not be present in some proteins), one or two zinc-finger domains, known as B boxes (B1 box and B2 box), and their associated coiled-coil region (3). RING domain confers E3 ubiquitin ligase activity to TRIM family proteins (4). TRIM proteins also contain a highly variable carboxyl (C) terminus, which can be divided into 11 subfamilies (C-I to C-XI) according to their C-terminal functional domains.

**Figure 1:**
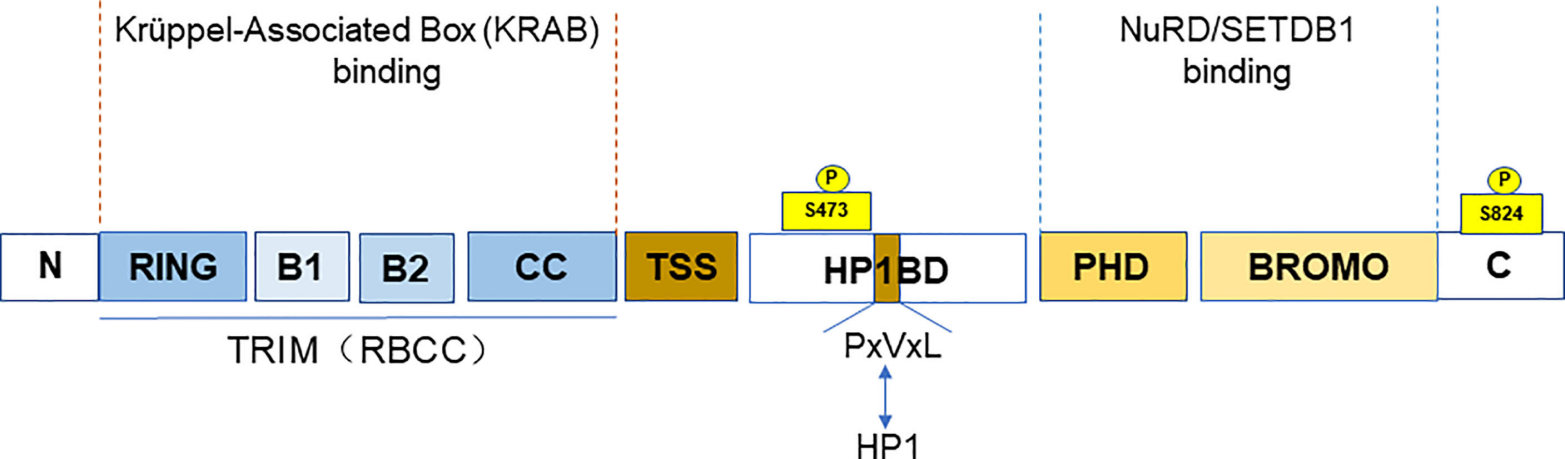
The domain and post-translational modifications of TRIM28. The figure shows that the different domains of TRIM28. A TRIM structure, including a RING finger, two B-boxes, and a leucine zipper coiled-coil region (CC) at the N-terminus. The PxVxL pentapeptide region and the central TIF1 signature sequence (TSS) are at the central part. And the plant homeodomain (PHD) finger and the Bromodomain are at the C-terminus. The TRIM domain mainly binds to the KRAB domain of KRAB-ZNFs. While PHD finger and bromodomain recruit NuRD and SETDB. The figure also shows the TRIM28 phosphorylation sites (S473 and S824).

TRIM28 is a member of the TRIM family, also known as KRAB (Krüppel-associated box)-associated protein 1 (KAP1) or transcriptional mediator 1β (TIF1β). TRIM28 (TIF1β), TRIM24 (TIF1α), TRIM33 (TIF1γ), and TRIM66 (TIF1δ) together form the transcriptional intermediary factor 1 (TIF1) family (5). TIF1 family has been reported to be abnormally expressed or mutated among the various cancers (6). TRIM28, along with TRIM24 and TRIM33, is classified as the C-VI subfamily of TRIM family proteins. At the N-terminus, TRIM28 contains a TRIM structure consisting of a RING finger, two B-boxes, and a leucine zipper coiled-coil region (CC). TRIM28 has E3 ubiquitin ligase activity due to the RING domain (7), which has the ability to link target proteins and recognize ubiquitin-loaded E2 conjugating enzyme, playing an important role in cellular protein degradation (4). The central part of TRIM28 contains the PxVxL pentapeptide region and the central TIF1 signature sequence (TSS). In addition, the plant homeodomain (PHD) finger and the bromodomain are at the C-terminus of TRIM28 (8). It is a nuclear protein and a general transcriptional co-repressor of the Krüppel-Associated Box Zinc Finger Protein (KRAB-ZFP) family. TRIM structure mediates the interactions with KRAB domains of KRAB-ZFP transcription factors. When TRIM28 is recruited by KRAB-ZNFs, it acts as a scaffold for a set of chromatin modification complexes. The PHD finger and bromodomain recruit the histone deacetylase complex (NuRD) and SET domain bifurcated histone lysine methyltransferase 1 (SETDB1). While the central PxVxL pentapeptide region binds Heterochromatin Protein 1 (HP1). All of these interactions are associated with heterochromatin formation and lead to transcriptional repression (9–11). The PHD finger–bromodomain also functions as a small intramolecular ubiquitin-like modifier (SUMO) E3 ligase in transcriptional silencing (12) (**Figure 1**).

**Figure 2:**
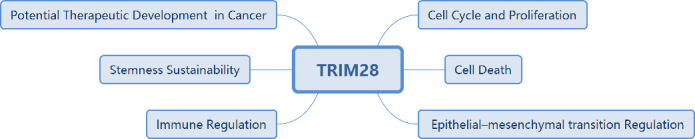
Biological function of TRIM28.

In addition to regulating gene transcription, TRIM28 has a variety of intracellular regulatory functions, including regulating the response to DNA damage and maintaining the stem cell pluripotency (13, 14). The relationship between TRIM28 and cancer development has been gradually reported. TRIM28 protein expression levels are elevated in cancers such as gastric, lung, cervical, breast and prostate cancers (15–18). High expression of TRIM28 is positively associated with poor prognosis in some cancers. Therefore, it is crucial to systematically explore the role of TRIM28 in tumorigenesis and progression. In this review, we summarized the important role of TRIM28 in tumorigenesis and development from five aspects, including tumor cell proliferation, tumor cell death, epithelial-mesenchymal transition (EMT), stemness, and immune microenvironment, and discussed the possibility of TRIM28 as a new tumor target (**Figure 2**).

## 2 Role of TRIM28 in the cancer cell cycle and proliferation

One of the best-known tumor suppressor, TP53, encodes the p53 protein, which is frequently deleted or inactivated by mutations in cancer (19, 20). As ubiquitin E3 ligase, mouse double minute 2 (MDM2) is a major regulator of p53. MDM2 binds p53 and promotes the ubiquitination of p53, which is the main mechanism for regulating the level of p53 in cells (21, 22). In lung cancer, RLIM can interact with MDM2 and degrade MDM2 by ubiquitination. TRIM28 can interact with RLIM and promote its ubiquitination, thereby maintaining the low expression level of p53 and ultimately promoting tumor cell proliferation and survival (15). In addition to regulating tumor cell proliferation by modulating p53, TRIM28 can also promote cervical cancer cell growth by activating the mammalian target of rapamycin (mTOR) signaling pathway. MTOR is important for the regulation of cell cycle, cell growth and proliferation (23). These cell proliferation could be abrogated by the specific mTOR inhibitor everolimus (24). Similarly, TRIM28 can promote endometrial cancer cell growth and migration by activating AKT/mTOR signaling pathway (25). TRIM28 can also play a role in tumor cell proliferation as a direct target of microRNAs. For example, miR-491 was under-expressed in glioblastoma multiforme tissues. And the downregulation of miR-491 can increase the expression of TRIM28 to promote glioma cell proliferation (26). In addition, miR-140-3p inhibits breast cancer cell proliferation and migration by directly regulating the expression of TRIM28 (27).

**Figure 3:**
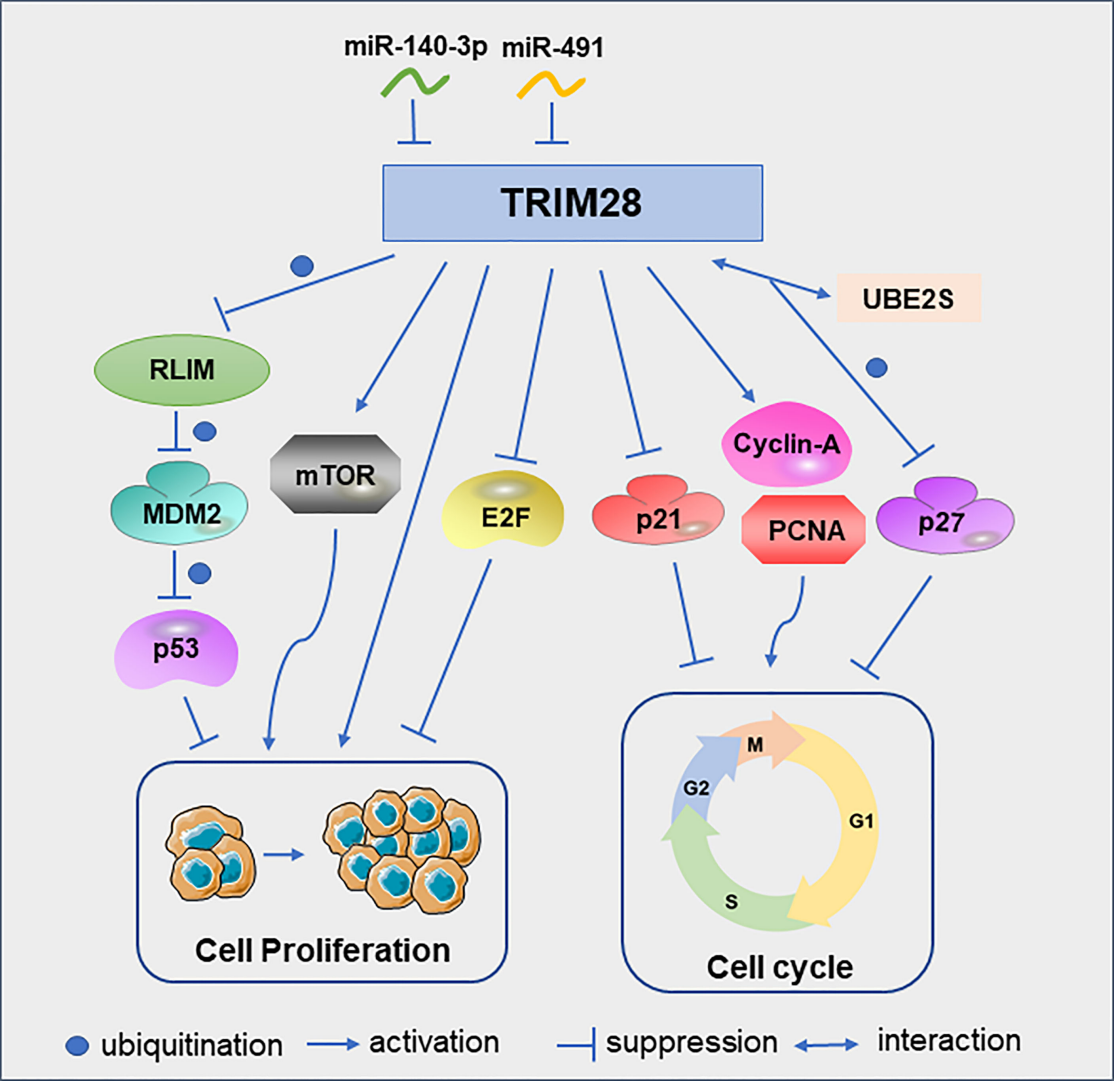
TRIM28 participates in the cell cycle and proliferation. Schematic diagram of the TRIM28 signaling pathway and effectors.

In B-cell non-Hodgkin lymphoma cells, TRIM28 can significantly increase the protein levels of cyclin A and PCNA, and reduce the protein level of p21, thereby promoting the cell cycle progression and cell proliferation (28). In glioma cells, down-regulation of TRIM28 increased the expression of p21 and induced cell cycle arrest in G1 phase (29). UBE2S, overexpressed in hepatocellular carcinoma(HCC), interacted with TRIM28 in the nucleus to jointly enhance the ubiquitination of p27 and promoted its degradation and cell cycle progression (30). Although the above studies discussed the promoting effect of TRIM28 on tumor cell proliferation, TRIM28 can also play an anti-proliferative role in early lung cancer by inhibiting the transcriptional activity of the E2F family (31) (**Figure 3**). The reason why TRIM28 has dual effects on tumor proliferation needs to be further explored.

## 3 Role of TRIM28 in cancer cell death

Cell death is closely related to the treatment of cancer (32). PCD includes apoptosis, autophagy, necroptosis, pyroptosis and ferroptosis (33). The interaction between TRIM28 and MDM2 plays a role in suppressing apoptosis of cancer cell. TRIM28 and MDM2 cooperate to suppress p53 acetylation, stimulate p53 ubiquitination, suppress p53 transcription and ultimately inhibit the apoptotic of tumor cells (34). The MAGE protein, which is upregulated in many cancer types, can act as a corepressor of p53 by binding to TRIM28 and inhibit p53-mediated apoptosis in MAGE-positive melanoma cell lines (35). MAGEA3 promotes proliferation and inhibits apoptosis in cervical cancer cells by suppressing the TRIM28/p53 signaling pathway (36). The ATM and p53-associated KZNF protein (Apak) attenuates p53 acetylation by recruiting TRIM28 and histone deacetylase 1 (HDAC1), thereby suppressing p53-mediated apoptosis (37). In addition, knockdown of TRIM28 in non-small cell lung cancer (NSCLC) resulted in a decrease in the anti-apoptotic gene B cell lymphoma (Bcl)−2 and an increase in the pro-apoptotic genes Bcl−2 associated X (Bax) and p53 (38). TRIM28 also can negatively regulate the expression of the pro-apoptotic genes Bcl-2-related ovarian killer (BOK) at the post-transcriptional level (39).

TRIM28 can also regulate the necroptosis of tumor cell. Necroptosis is thought to be a type of cell death that increases tumor immunogenicity (40). Phosphorylation of TRIM28 at serine 473(S473) plays a crucial role in necroptosis. In necroptosis cells, activated RIPK1 kinase mediates the formation of the RIPK1/RIPK3/MLKL complex, which promotes MLKL oligomerization and necroptosis. Oligomeric MLKL-mediated activation of p38 MAPK promotes phosphorylation of TRIM28 at S473, which in turn mediates the inflammatory response during advanced necroptosis (41). Activation of RIPK3 promotes phosphorylation of TRIM28 at S473, which in turn inhibits the chromatin-binding activity of TRIM28, leading to the transcriptional activation of cytokines, and ultimately promoting immunomodulatory processes such as dendritic cell maturation and further cytokine production, thereby promoting anticancer response (40).

**Figure 4:**
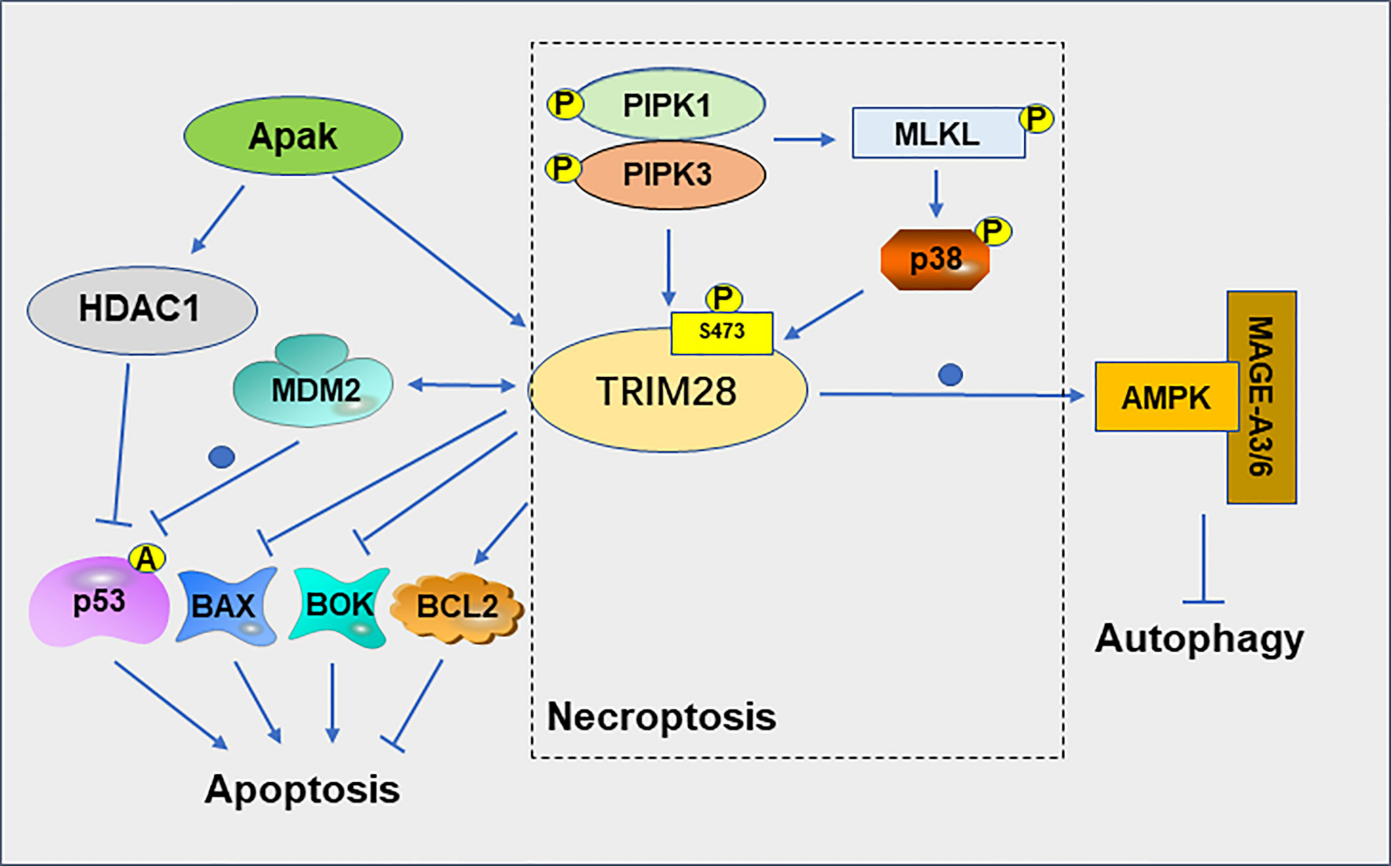
Role of TRIM28 on apoptosis, necroptosis and autophagy.

Autophagy plays different roles in different stages of tumor development. For example, autophagy generally inhibits tumor progression in the early stages of cancer development, but promotes tumor growth in the late stage (42). TRIM28 is required for the production of inosine phosphate and the formation of autophagic vesicles (AVs) in autophagy (43). TRIM28 is also involved in the regulation of mitophagy, a selective degradation of mitochondria through autophagy (44). In gliomas, the expression level of TRIM28 and autophagy levels are significantly elevated with the progression of tumor grade. Downregulation of TRIM28 can inhibit the autophagy of glioblastoma cells (45). TRIM28 can degrade the cellular energy sensor and regulator AMP-activated protein kinase (AMPK) by ubiquitination pathways, resulting in significantly reduced autophagy, altered cellular metabolism, and activation of mTOR signaling (46) (**Figure 4**). MAGE-TRIM28 axis also affects the Warburg effect and HCC progression by targeting fructose-1,6-biphosphatase (FBP1) and promoting its degradation, suggesting that MAGE-TRIM28 axis regulates the metabolic reprogramming of cancer cells by modulating the protein degradation of various metabolic regulators (47). It can be seen from above that TRIM28 plays a crucial role in tumor cell apoptosis, necroptosis, and autophagy, but the relationship between TRIM28 and pyroptosis and ferroptosis has not been reported. Whether TRIM28 plays a role in these two modes of death requires further investigation.

## 4 Role of TRIM28 in epithelial–mesenchymal transition

**Figure 5:**
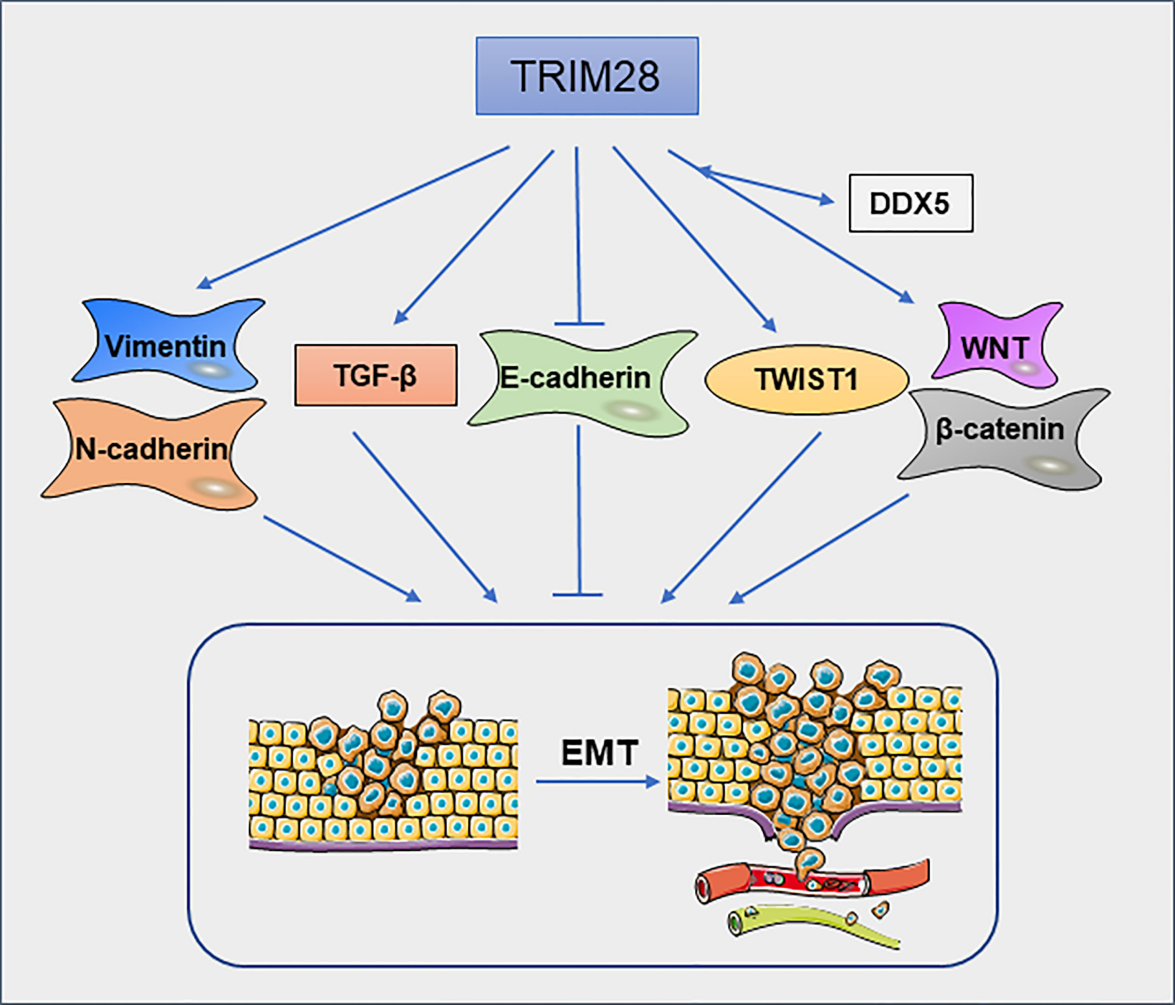
TRIM28 regulates epithelial–mesenchymal transition. TRIM28 promotes EMT and leads to enhanced migration and invasion of tumor cells.

Epithelial–mesenchymal transition (EMT) is a cellular process in which cells lose epithelial features and gain mesenchymal characteristics. EMT is closely related to tumor cell migration and invasion (48). Christo D. Venkov et al (49) found that a ternary complex formed by TRIM28, the CArG box–binding factor-A (CBF-A) and fibroblast transcription site-1 (FTS-1) can induce the expression of fibroblast-specific protein 1(FSP1), which is a new EMT proximal activator. In addition, overexpression of TRIM28 significantly decreases the expression of epithelial marker E-cadherin and increased the expression of mesenchymal markers vimentin and N-cadherin in pancreatic cancer(PC) cells (50). These findings suggest that TRIM28 may promote metastasis of PC by regulating the EMT. In NSCLC cells (51), knockdown of TRIM28 can block TGF-β-induced EMT, thereby decreasing the tumor cell migration and invasion. Mechanically, the expression of TRIM28 can regulate the acetylation and methylation of histones on E-cadherin and N-cadherin promoter regions. Moreover, in breast cancer cells, TRIM28 can enhance EMT by stabilizing TWIST1 to promote breast cancer metastasis (52). TRIM28 deficiency decreases TWIST1, N-cadherin and upregulates E-cadherin protein levels, thereby inhibiting cell migration and invasion. TRIM28 is also involved in the metastasis of ovarian carcinoma (OC) cells. TRIM28 is required for the activation of β-catenin. Silencing of TRIM28 inhibits the migration, invasion and EMT process of OC cells *via* blocking Wnt/β-catenin signaling pathway (53). Moreover, in thyroid cancer, knockdown of TRIM28 inhibits P68/DEAD box protein 5(DDX5)-mediated Wnt/β-catenin signaling pathway (54) (**Figure 5**). In cervical cancer, ZBRK1 can inhibit the transcription of TRIM28, thereby regulating the expression of metastasis-related genes (16). TRIM28 is an independent factor for peritoneal dissemination of gastric cancer. TRIM28 promotes gastric cancer progression *via* its antiapoptotic effects, which leads to EMT (55). In Epstein–Barr virus (EBV)-associated gastric cancer, EBV-encoded small ribonucleic acid 1(BHRF1) upregulates the expression of SNHG8, which as a sponge of miR-512-5p, leading to increased expression of TRIM28, and promoting malignant progression of the tumor (56). TRIM28 can promote EMT in various types of cancer (**Figure 5**), but the specific mechanism still needs to be further explored. It is undeniable that TRIM28 may have an important effect on tumor migration and invasion by regulating EMT.

## 5 Role of TRIM28 in cancer cell stemness sustainability

Regulatory networks that control stem cell self-renewal may also be active in some cancers. TRIM28 and Cnot3 together with c-Myc and Zfx co-occupy many putative gene promoters in mouse embryonic stem (ES) cells. They form a distinct module in the self-renewing transcriptional network, independent of the core module formed by Nanog, Oct4 and Sox2. Most of the target genes of these four factors were down-regulated during ES cell differentiation, while transcriptional regulators involved in cell cycle, cell death and cancer were enriched (14). TRIM28, an Oct4-binding protein, prevents itch-mediated ubiquitination and subsequent Oct4 degradation (57). Furthermore, TRIM28 regulates Oct3/4–dependent transcription in a phosphorylation-dependent manner. In pluripotent mouse ES cells, phosphorylated TRIM28 at ser824 can specific induce the expression of ES cell-specific genes and keep the mouse ES cells in an undifferentiated states for a long time (58). In addition to inducing the expression of ES cell-specific genes, TRIM28 also repressed the expression of differentiation-inducing genes and pluripotency-related genes in ES cells (59). TRIM28 has also identified as an epigenetic barrier to the reprogramming of induced pluripotent stem cell (60, 61). Downregulation of TRIM28 causes the rapid loss of self-renewal capacity and immediate differentiation of nascent pluripotent stem cells (iPS cells), therefore TRIM28 is essential for stabilizing the formation of iPS cells. Moreover, TRIM28 utilizes KRAB- ZNFs to cause epigenetic silencing of its differentiation-related target genes by H3K9Me3 and DNA methylation in human pluripotent stem cells (62).

**Figure 6:**
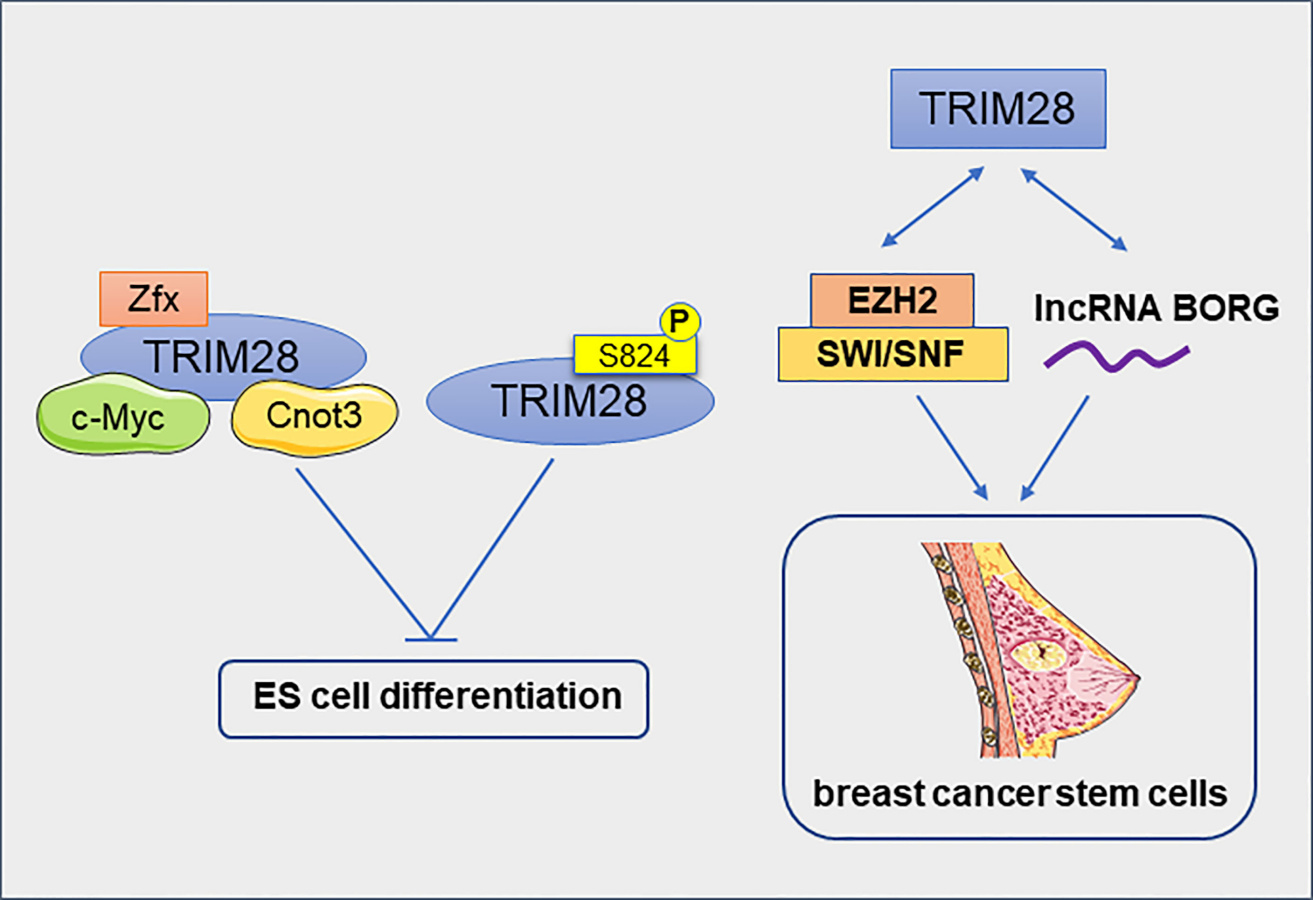
TRIM28 inhibits the differentiation of embryonic stem cells and promotes the enrichment and maintenance of breast cancer stem cells.

Cancer stem cells (CSCs) are found in many types of cancer. The CSC theory suggests that tumor growth is driven by a small amounts of CSCs hidden in the cancer (63). TRIM28 has been reported to play an important role in breast cancer stem cells (BCSCs). The reduction of TRIM28 gene expression reduced the self-renewal ability of BCSCs and resulted in a significant decrease in tumor growth (64). In estrogen receptor- positive breast cancer cell line MCF7, TRIM28 interacts with histone methyl transferase Enhancer of Zeste Homolog 2 (EZH2) and SWI/SNF to activate the expression of genes that promote CD44^hi^/CD24^lo^ mammosphere formation (65). TRIM28 interacts physically with lncRNA BORG to promote the development of BCSC. lncRNA BORG/TRIM28 complexes induced BCSC self-renewal and expansion *in vitro* and induced the metastatic outgrowth of BCSC in the lungs of mice (66). All of these results support that TRIM28 promotes the enrichment and maintenance of BCSCs (**Figure 6**). In addition, TRIM28 protein has also been reported to regulate the stem cell-like phenotype of melanoma. Melanoma cell lines with high TRIM28 expression were more likely to form melano-sphere. Melanoma with high TRIM28 expression was also significantly enriched c-Myc-related gene markers. This suggests that the melanoma stem-like phenotype of melanoma with high TRIM28 expression may be caused, at least in part, by significant activation of c-Myc (67). In Glioblastoma (GB), TRIM28 was also overexpressed in tumor stem cells and associated with the expression of stem cell-related genes (68). The expression of KRAB-ZNFs and TRIM28 were significantly negatively correlated with the dedifferentiation status of kidney renal clear cell carcinoma (KIRC) (69). Therefore, TRIM28 maybe a positive regulator of the CSCs. In conclusion, TRIM28 plays an important role in maintaining the pluripotent state of normal and cancer stem cells by inhibiting the expression of differentiation-related genes and inducing the expression of stem cell-related markers. Targeting TRIM28 may effectively prevent cancer metastasis by eliminating CSCs.

## 6 Role of TRIM28 in immune regulation

**Figure 7:**
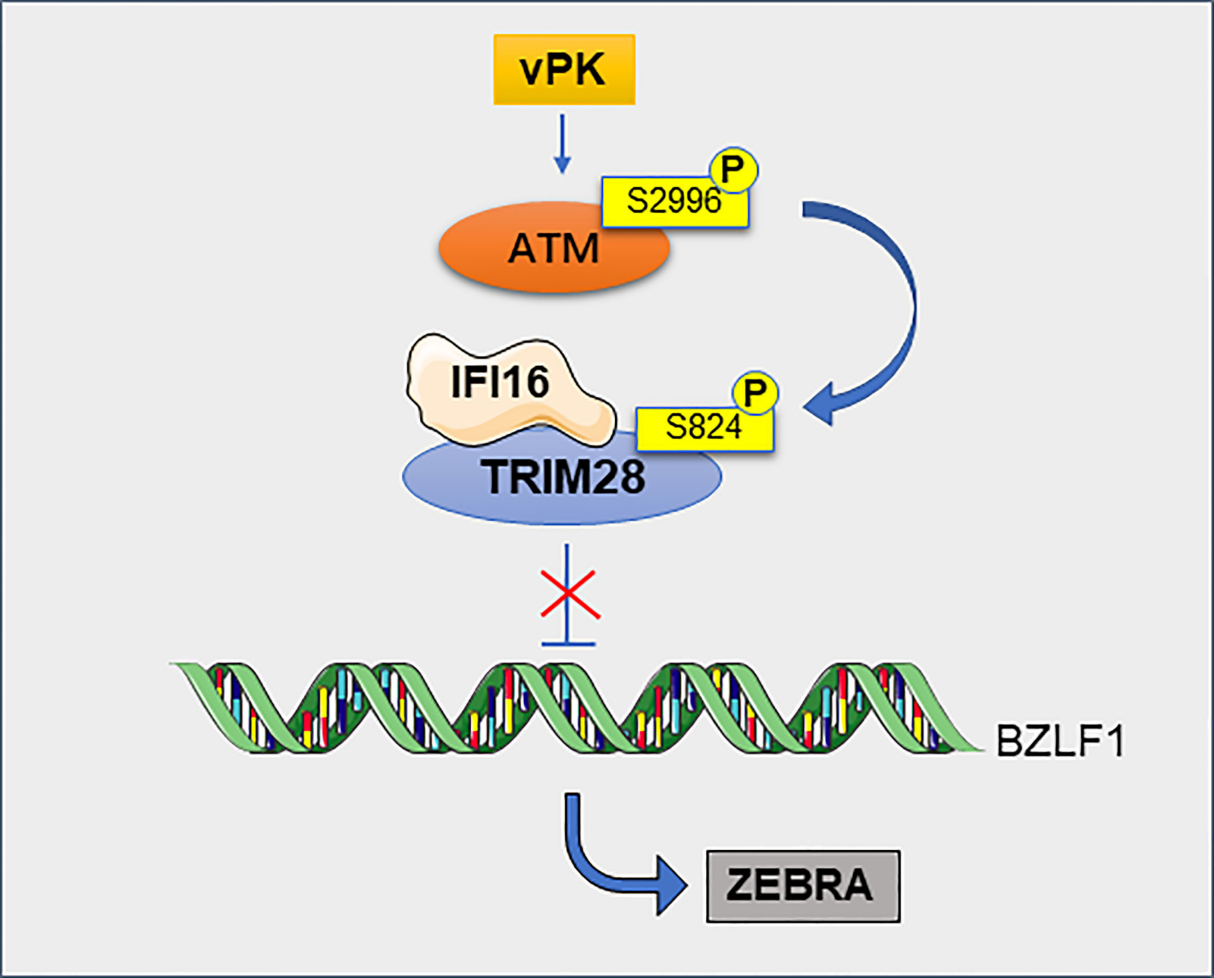
TRIM28 contributes to the maintenance of EBV latency. TRIM28 interacts with IFI16 and this cooperation silences the EBV cleavage switch protein ZEBRA encoded by the BZLF1 gene, thereby maintaining viral latency. Viral protein kinase (vPK) activates cellular phosphatidylinositol 3 kinase-associated kinase ATM *via* atypical site phosphorylation, leading to the phosphorylation of TRIM28, this phosphorylation making it impossible to inhibit ZEBRA and ultimately to viral production.

Viral infection is closely related to tumorigenesis. Herpesviruses are able to persist for a long time in infected hosts without producing viral particles (70). TRIM28 plays an important role in herpes virus-associated tumors such as EBV, Kaposi’s sarcoma-associated herpesvirus (KSHV) and human cytomegalovirus (HCMV) (71). EBV infects more than 95% of adults worldwide and is associated with the development of a variety of tumors, including nasopharyngeal and gastric cancers (72). KSHV is the causative agent of Kaposi’s sarcoma (73). TRIM28 can maintain the latent state of EBV and KSHV by binding to the promoters of viral genes (71). TRIM28 is a target of latent membrane protein-1 (LMP1)-induced sumoylation, which helps to the maintenance of EBV latency (74). The latest study shows that TRIM28 can interacts with interferon-gamma inducible protein 16 (IFI16) to silence the expression of EBV lytic genes (75). Notably, phosphorylation of TRIM28 blocks the inhibitory function of TRIM28 on virus latency, resulting in virus production (76) (**Figure 7**). Likewise, in latently infected hematopoietic stem cells, TRIM28 recruits HP1 and SETDB1 to the HCMV genome, resulting in transcriptional silencing. When phosphorylated at serine 824, TRIM28 promotes viral maintenance at latency, which prevents its ability to recruit SETDB1 and thus abrogated its inhibitory potential (77). Cancers are now known to be associated with human immunodeficiency virus type 1 (HIV-1) (78). TRIM28 potently inhibits HIV-1 gene expression by exploiting SUMO E3 ligase activity and epigenetic adaptor function (79). TRIM28 is also a repressor of endogenous retroviruses (ERVs) (80). In embryonic carcinoma (EC) and embryonic stem (ES) cells, TRIM28 binds to the primer binding site (PBS) of moloney murine leukemia virus (M-MLV) and repress transcription of the viral promoter, leading to restriction of M-MLV replication (81). Endogenous retroviral activation is a key mechanism of antitumor immune responses in radiotherapy. TRIM28 is a potential therapeutic target to enhance radiation-induced antitumor immune responses (82).

The SETDB1-TRIM28 complex is a major suppressor of antitumor immunity (83). Inhibition of SETDB1-TRIM28 upregulates the expression of programmed cell death ligand 1 (PD-L1) and activates the circulating GMP-AMP synthase (cGAS)-stimulator of interferon gene (STING) innate immune response pathway to increase the infiltration of CD8+ T cells. Melanoma with high TRIM28 expression showed less immune cell infiltration, including Cytotoxic T cells, helper T cells, B cells, macrophages, and eosinophils (67). Moreover, IRF transcription factor family members (IRF1, IRF2, IRF5, IRF8) were significantly down-regulated in melanoma with high TRIM28 expression, which corresponded to the weakening of interferon signaling. It was previously reported that TRIM28 is an interaction partner of IRF1 and can negatively regulate STAT1-mediated IRF1 expression (84). In addition, TRIM28 is a specific SUMO E3 ligase for IRF7 (85). TRIM28 interacts with the N-terminus of IRF5 and inhibits its function in inflammatory macrophages (86).

TRIM28 is also involved in T cell development and activation. In cancer, the presence of T cells (Tregs) hinders the development of effective antitumor immunity in tumor patients (70). The transcriptional regulator FOXP3 is essential for Treg development and function. TRIM28 can interact with FOXP3 in human Tregs. In addition, the expression of Slc1a5 was significantly reduced in TRIM28-deficient Tregs, which resulted in inhibition of mTORC1 activation thereby inhibiting the Tregs proliferation and activation. Thus, TRIM28 can regulate the function and proliferation of Treg in a FOXP3-dependent and-independent manner. Another study showed that TRIM28 controls metabolic reprograming by epigenetic silencing of a defined set of Treg-characteristic genes, promoting effective T cell expansion and differentiation into helper and regulatory phenotypes (87). Expression of TRIM28 on dendritic cells prevents T cell over-priming by silencing ERVs (88).

In conclusion, TRIM28 helps maintain the virus in a latent state and suppresses the generation of anti-tumor immune environment. However, the role and mechanism of TRIM28 in tumor immunity still needs to be further explored.

## 7 Prospect of TRIM28 as a potential target in cancer treatment

Cytotoxic anticancer drugs and targeted drugs play an important role in clinical cancer treatment. However, the drug resistance of tumor cells seriously affects the anti-cancer effects of drugs (89). Knockdown of TRIM28 significantly increases the sensitivity of lung cancer cells to 5-FU. The resistance of lung cancer cells to 5-FU may be related to the role of TRIM28 in promoting cell proliferation (90). Bortezomib (BTZ), a selective proteasome inhibitor, has shown promising in the treatment of HCC, but drug resistance remains a major problem hinders its efficacy. Notably, TRIM28 attenuates BTZ sensitivity to hepatocellular carcinoma cells by enhancing proteasome expression (91). TRIM28 expression is highly upregulated in castration resistant prostate cancers (CRPC). TRIM28 overexpression enhances AR signaling, which is a key mechanism for anti-androgen deprivation therapy in prostate cancer (17). Conversely, BCL2A1 contributes to chemotherapy resistance in tumor subpopulations, As an E3 ubiquitin ligase of BCL2A1, TRIM28 overexpression reduces BCL2A1 protein levels and restores the sensitivity of melanoma cells to BRAF-targeted therapy (92). E2F1 is a common target for the development of anticancer chemotherapeutics such as etoposide and doxorubicin. However, tumor cells are insensitive to chemotherapeutic drugs that target E2F1 activation (93). This may be related to the expression of TRIM28, which recruits HDAC1 to deacetylate E2F1, thereby inhibiting the activity of E2F1. Moreover, knockdown of TRIM28 increases etoposide-induced cell death (94). Phosphorylation of TRIM28 at ser473 after DNA damage enhances its interaction with E2F1. Combination chemotherapy with etoposide and an appropriate TRIM28 S473p-blocking peptide may help to induce tumor cell death. The p53 pathway is also a major target for cancer drug development (95). In TRIM28-depleted cells, actinomycin D treatment promoted p53 activation, suggesting that TRIM28 may be a target of p53 reactivation in cancer cells. Treatment of cells with actinomycin D with compounds that inhibit the TRIM28-Mdm2 interaction or reduce TRIM28 levels, can effectively inhibit the function of Mdm2, resulting in strong activation of p53 (96). Reactive oxygen species (ROS) can achieve therapeutic purposes by accelerating tumor cell death (97). Peroxide-induced p38 MAPK mediates the phosphorylation of TRIM28 at S473 and TRIM28 activation, which effectively promotes DNA repair to assist tumor cells to survive against exogenous ROS (98). Therefore, inhibiting of TRIM28 may promote tumor cells to be more sensitive to chemotherapy.

Various nanobodies have been used in clinical trials to treat a variety of diseases (99). The anti-TRIM28 selective nanobody NB237 significantly inhibits the invasion and metastasis of Glioblastoma (GB) cells and GB stem cells in the zebrafish brain (68). In cardiomyocytes, it has been reported that Zenglv Fumai Granule (ZFG) protect cardiomyocytes against hypoxia/reoxygenation-induced apoptosis by inhibiting TRIM28 expression (100). There is a need to develop small molecule inhibitors targeting TRIM28, which may be an adjunct to tumor therapy. Additionally, TRIM28 is a transcriptional activator of the mutant telomerase reverse transcriptase (TERT) promoter in bladder cancer (BC). Inhibition of mTORC1 with rapamycin analog Ridaforolimus inhibits TRIM28 phosphorylation, hTERT expression, and cell viability (101). PD-L1 is a key driver of tumor-mediated immunosuppression, and verteporfin is a major small molecule inhibitor of PD-L1. Studies have shown (102) that verteporfin can inhibit basal and interferon (IFN)-induced PD-L1 expression *in vitro* and *in vivo* through targeting Golgi-associated autophagy and interfering with the STAT1-IRF1-TRIM28 signaling cascade.

Taken together, TRIM28 may be responsible for drug resistance during the treatment of certain cancers, and is also an important factor that causes some anticancer drug targets to be insensitive to drugs. The purpose of promoting tumor treatment can be achieved by developing some drugs or small molecules that specifically target TRIM28. This is exactly what is lacking in the current research on TRIM28.

## 8 Discussion

The role of TRIM28 in tumorigenesis and progression are diverse. TRIM28 may act as an oncogene to promote the proliferation and survival of cancer cells and promote EMT to enhance the migration and invasion ability of cancer cells. In terms of immune regulation, TRIM28 is considered to be a negative immune regulator. The role of TRIM28 in cancer stem cells also suggests a tumor-promoting function. However, TRIM28 may function as a tumor suppressor. TRIM28 plays a critical role in maintaining the intrinsic phenotype of non-tumor cells without malignant transformation.

In conclusion, although TRIM28 is often described as an oncogene, it may have dual roles as an oncogene or a tumor suppressor, and further research is needed to clarify the exact role of TRIM28 in the occurrence and development of cancer. TRIM28 often shows abnormal gene expression in cancer, which provides the possibility to target it for anticancer therapy. TRIM28 may also be an attractive pharmacological target for enhancing the effects of radiotherapy and immunotherapy in cancer therapy. It is undeniable that based on the important role of TRIM28 in tumorigenesis and development, TRIM28 may become a new target for targeted therapy in the future, providing new strategies for disease prevention, diagnosis and treatment.

## Author contributions

YY, ST drafted the manuscript and prepared the figures. YH, LH, RY, ZH, YiT, LO, JL, QP, XJ, XX, LX, MP, NW and YaT helped in collecting the related literatures and participated in discussion. XL, QL and YZ designed the review and revised the manuscript. All authors contributed to the article and approved the submitted version.
